# Variation in symbiotic N_2_ fixation rates among *Sphagnum* mosses

**DOI:** 10.1371/journal.pone.0228383

**Published:** 2020-02-04

**Authors:** Eva van den Elzen, Fia Bengtsson, Christian Fritz, Håkan Rydin, Leon P. M. Lamers

**Affiliations:** 1 Department of Aquatic Ecology and Environmental Biology, Institute for Water and Wetland Research, Radboud University, AJ Nijmegen, the Netherlands; 2 Department of Plant Ecology and Evolution, Evolutionary Biology Centre, Uppsala University, Uppsala, Sweden; Chinese Academy of Sciences, CHINA

## Abstract

Biological nitrogen (N) fixation is an important process supporting primary production in ecosystems, especially in those where N availability is limiting growth, such as peatlands and boreal forests. In many peatlands, peat mosses (genus *Sphagnum*) are the prime ecosystem engineers, and like feather mosses in boreal forests, they are associated with a diverse community of diazotrophs (N_2_-fixing microorganisms) that live in and on their tissue. The large variation in N_2_ fixation rates reported in literature remains, however, to be explained. To assess the potential roles of habitat (including nutrient concentration) and species traits (in particular litter decomposability and photosynthetic capacity) on the variability in N_2_ fixation rates, we compared rates associated with various *Sphagnum* moss species in a bog, the surrounding forest and a fen in Sweden. We found appreciable variation in N_2_ fixation rates among moss species and habitats, and showed that both species and habitat conditions strongly influenced N_2_ fixation. We here show that higher decomposition rates, as explained by lower levels of decomposition-inhibiting compounds, and higher phosphorous (P) levels, are related with higher diazotrophic activity. Combining our findings with those of other studies, we propose a conceptual model in which both species-specific traits of mosses (as related to the trade-off between rapid photosynthesis and resistance to decomposition) and P availability, explain N_2_ fixation rates. This is expected to result in a tight coupling between P and N cycling in peatlands.

## Introduction

Nitrogen (N) fixation is the only biological pathway in which atmospheric dinitrogen (N_2_) is converted to a reduced form (NH_x_) accessible to plants. In this way N_2_-fixing microorganisms (diazotrophs) significantly contribute to the N pools of ecosystems, and thereby affect primary production of plants, given that N availability is often limiting plant biomass production [1]. Knowledge of the drivers of the strongly varying rates of N_2_ fixation reported in literature is therefore vital for our understanding of the relative contribution of N_2_ fixation to the total ecosystem N input [2, 3]. Human interference through the development of techniques to artificially fix N_2_ for agricultural production has led to extensive eutrophication, disrupting the global N cycle [4, 5]. As this disturbance of the natural N input into ecosystems also affects carbon (C) cycling through its effects on primary production and decomposition [6, 7], knowledge about the drivers of N_2_ fixation becomes even more important.

The relative contribution of N_2_ fixation to the total available N pool can be expected to be most significant in those ecosystems, such as *Sphagnum* peatlands, where atmospheric N deposition represents the only other major N input. The low N deposition at northern latitudes, in combination with the competitive strategy of *Sphagnum* mosses to sequester N, speaks for the importance of N_2_ fixation in these habitats. In pristine boreal peatlands, which are long term C sinks [8, 9], rates of N_2_ fixation in *Sphagnum* were indeed found to explain the discrepancy between the low N inputs through atmospheric deposition, and the N assimilation of *Sphagnum* species and N storage in peat [10]. In *Sphagnum* peatlands the contribution of diazotrophic N_2_ fixation is estimated to constitute around 35% of the N input [11, 12], while in N-limited boreal forests, N_2_ fixation in mosses was found to contribute up to 50% to the total N input [13].

Peat mosses (genus *Sphagnum*) are the prime ecosystem engineers of many peatlands, hampering other plants’ growth for example by strongly monopolising N input from atmospheric deposition [14–16]. Also, *Sphagnum* deters competitors and decomposers through waterlogging, acidification and the production of recalcitrant organic compounds and structurally rigid cells: characteristics that enable them to produce and store large amounts of peat over time [16, 17]. Functional traits of *Sphagnum* mosses, such as litter decomposability, growth and acidifying potential, are in large part due to the biochemistry of the mosses [18, 19], and their traits affect ecosystem processes such as C sequestration in *Sphagnum* dominated peatlands [20]. N_2_ fixation potential represents an important functional trait that contributes to ecosystem N input. In this way plant-associated microbiomes explain additional variation in productivity and ecosystem functioning [21, 22].

*Sphagnum* mosses are colonised by a diverse community of microorganisms that live inside the large volume of dead hyaline cells and on the surface of their tissue [23, 24]. The community may comprise a high proportion (45.5%; [25]) of highly diverse diazotrophs that profit from the moist and partly anoxic conditions around and in *Sphagnum*. Other studies have shown that most of the N_2_-fixing activity in *Sphagnum* was assigned to the Alphaproteobacterial class of the phylum Proteobacteria, and only very little (6% or less) to the phylum Cyanobacteria [25–27]. The ratio between Proteobacteria and Cyanobacteria can, however, vary among peatlands [28]. This microbiome composition is in stark contrast to that of feather mosses that dominate the forest floor of boreal forests. The feather moss *Pleurozium schreberi* was associated with diazotrophs whose genes were 96% cyanobacterial [29]. Much research has focused on the differential rates of N_2_ fixation of cyanobacteria in these feather mosses, and its inhibition by increasing N deposition [29–31]. However, for *Sphagnum*, much less is known about the variation in N_2_ fixation rates in different species and habitats, and how environmental drivers control these rates.

Different *Sphagnum* species were found to have different microbial communities [24], resulting in differences in N_2_ fixation rates [27]. At the same time, different *Sphagnum* species are also adapted to the different environmental conditions of their habitats [32, 33], which can also be expected to have an effect on the composition and activity of the diazotrophic community [28]. For example, N_2_ fixation rates in *Sphagnum* species of fens were found to be higher than in *Sphagnum* species in bogs [34]. This may be related to high pH [35] or higher phosphorus (P) availability [36]. In bogs, rates were found to be higher in hollows compared to hummocks [37], suggested to be the result of water level [27]. Especially the effect of P, important for the synthesis of ATP necessary for the costly biochemical process of N_2_ fixation, is considered to be important in driving N_2_ fixation rates in terrestrial ecosystems in general [38], and in feather mosses [39] and *Sphagnum* [36].

Here, we study the variation in N_2_ fixation rates among host species and habitats, by comparing four *Sphagnum* mosses in contrasting habitats and relating N_2_ fixation to nutrient levels in the mosses. Specifically, we relate the N_2_ fixation to species traits associated with growth and decomposition. Synthesising our results with those of other studies, we generate a conceptual model that illustrates the interconnected drivers of N_2_ fixation and *Sphagnum* performance in mires. In addition, we compare the N_2_ fixation rates in *Sphagnum* with two well-studied boreal feather mosses.

## Material and methods

### Species and study sites

Our study sites are located in the central-east of southern Sweden. Kulflyten mire (59°54’N, 15°50’E) is a raised ombrotrophic bog with bog pools, surrounded by a wet lagg fen that is richer in solutes because of the surrounding mineral soil. The mire is further surrounded by a young spruce forest on peaty soil. The second site, Glon (60°31’N, 17°55’E) is a small rich fen on lime-rich moraine. The pH in the bog among our samples averages 4.3 (±0.02), while it is 5.5 (±0.03) around the *Sphagnum* sampled in the rich fen. Both sites show a mean temperature of around 17°C in July, and –2.6°C (Kulflyten) and –1.0°C (Glon) in December [32]. No specific permissions were required at these sites because Swedish public access to land allows everyone to access nature areas and collect plants, as long as an area does not have a protected status which states that you can not collect plants. These areas did not have protected status prohibiting collection of moss or other plants. The field study did not involve endangered or protected species.

Our sampling aimed to explore the variation among species and habitats. At Kulflyten mire we sampled four different vegetation types: open bog (OB), pine bog (PB), spruce forest (SF) and lagg fen (LF). At the fen, Glon, one habitat type was sampled: rich fen (RF). We sampled four species of *Sphagnum*: *S*. *fallax* (LF), *S*. *fuscum* (OB, RF), *S*. *rubellum* (OB), *S*. *magellanicum* (OB, PB, SF). Additionally, we sampled two species of feather mosses: *Pleurozium schreberi* (SF) and *Hylocomium splendens* (SF) (Table 1).

**Table 1:** Species used in the study, vegetation types and microtopographical positions along the hummock-hollow gradient.

Species	Author	*Sphagnum* subgenus	Vegetation type	Micro-topographical position
*Sphagnum fuscum*	(Schimp.) H.Klinggr.	*Acutifolia*	Open bog (OB) Rich fen (RF)	Hummock Hummock
*Sphagnum rubellum*	Wilson	*Acutifolia*	Open bog (OB)	Low hummock
*Sphagnum fallax*	(H.Klinggr.) H.Klinggr.	*Cuspidata*	Lagg fen (LF)	Lawn
*Sphagnum magellanicum*	Brid.	*Sphagnum*	Open bog (OB) Pine bog (PB) Spruce forest (SF)	Lawn–carpet Hummock Hummock
*Pleurozium schreberi*	(Brid.) Mitt.	-	Spruce forest (SF)	-
*Hylocomium splendens*	(Hedw.) Schimp.	-	Spruce forest (SF)	-

### Sampling and N_2_ fixation measurements

Samples were collected in September 2014 in homogenous patches of single species. For each species, five replicates (four for *S*. *magellanicum*, SF), from similar patches were obtained. For *Sphagnum* these are the patches where we measured several traits, including photosynthetic capacity, in the previous study [32, 40]. Within each replicate three subsamples were taken. A sharpened metal ring (ø 5 cm, depth 5 cm) was carefully inserted into the vegetation without compressing it, by making a pre-cut circle using scissors. Samples were put in plastic ziplock bags and directly transported to the lab in Nijmegen, the Netherlands. Two of the subsamples were dried for 72 h at 70° C and weighted to determine dry weight (DW) per area and bulk density (g cm^-3^). These subsamples were homogenised and ground with a mixer mill (MM301, Retsch, Germany) for 2 min at 30 rotations s^-1^ and used as control samples to determine background isotopic N signature and tissue nutrient concentrations.

The third subsample of each species-habitat combination replicate was put in a 120 ml flask with a capped rubber stopper. With an injection needle, 12 ml of the headspace air was removed and replaced with ^15^N_2_ gas (98 atom % ^15^N, Sigma-Aldrich, Germany) resulting in a headspace concentration of 10% of ^15^N_2_. The mosses were incubated for 48h in an incubation room (light regime 16h per day, 200 μmol m^-2^ s^-1^ PAR, 20°C). After incubation, mosses were dried and ground (see above). For both background and enriched samples, total N concentrations, isotopic ratios (^15^N/^14^N, expressed with δ^15^N signal as the ‰ deviation from atmospheric N_2_) and atom percent ^15^N/^14^N (^15^N) were determined using an elemental analyser (Type NA 1500 Carlo Erba, Thermo Fisher Scientific Inc, USA) coupled online via an interface (Finnigan Conflo III) to a mass spectrometer (Thermo Finnigan DeltaPlus, USA). The isotope atom percent ^15^N of the enriched samples was corrected by the natural isotopic ^15^N abundance of the background samples by subtracting enriched atom% ^15^N with natural atom% ^15^N to get atom% increase in ^15^N. This atom% increase in ^15^N is converted to g N gDW^-1^ with the total %N according to Eq 1.

$$N_{2}\;fixation=\frac{atom\%\;increase\;15N}{100\%}\times \frac{total\;\%N}{100\%}\times \frac{total\;\%N\;in\;atmosphere}{\%\;15N2\;in\;headspace}$$ (1)

N_2_ fixation rates in g N gDW^-1^ were converted to rates in nmol N_2_ gDW^-1^ d^-1^ using incubation time and molecular weight. Light regime during incubation was representative for the average day length of the growth season (16 hours), and the short period of raising the temperature to 20°C was expected to have little effect on N_2_ fixation rates [41, 42].

Total carbon (C) concentration was determined using an elemental analyser (see above) and total phosphorus (P) and potassium (K) concentrations were determined on digestates of dried and ground moss tissue, which were prepared by digesting samples in 500 μL HNO_3_ (65%) and 200 μL H_2_O_2_ (30%) for 16 min in a microwave (Milestone MLS1200 Mega, Milestone Inc., Sorisole, Italy). Digestates were diluted with MilliQ water (PFXXXXM1, Elga, UK) and P and K concentrations measured by inductively coupled plasma emission spectrometry (IRIS Intrepid II, Therma Electron corporation, Franklin, USA).

### *Sphagnum* performance and traits

We used data on *Sphagnum* performance and environmental variables that were collected at the same sample patches and analysed by Bengtsson et al. [32, 40], where methods and data are described in detail. We included data on biomass accumulation per unit area, CO_2_ exchange rate per unit biomass, decomposition in the field and standardised decomposability in the lab (% mass loss from litter). Biomass accumulation was averaged over the two growing seasons 2013 and 2014. Length increment was measured along brush wires inserted into the *Sphagnum* vegetation, as described in Rydin and Jeglum [16]. Biomass accumulation was determined by counting the shoot density in cores (ø = 7 cm) and multiplying the DW of 3 cm shoot-sections with the length increment. Photosynthetic capacity was measured as the maximum net CO_2_ exchange rate at optimal water content and expressed per individual shoot.

Moss decomposability was measured in samples from the same patches used by Bengtsson et al. [19, 43], where methods and data are described in detail. Decomposability of the litter was determined as mass loss after litter had been incubated in the lab for 7 months. Litter was defined as 2 cm shoot sections from below the capitula, which was placed in nylon mesh bags. To estimate field mass loss, a second set of litterbags was buried in the field at the original location of each bag, 5 cm below the moss surface. Mass loss was assessed after 14 months. We also use data for the same *Sphagnum* patches on biochemical composition of the litter: sphagnan, soluble phenolics and lignin-like phenolics (all in mg g^-1^).

### Statistical analysis

To analyse whether rates of N_2_ fixation varied between species and habitats we used ANOVA. Samples of *S*. *fuscum* and *S*. *magellanicum* from different habitats were treated as different units, i.e. on the same level as species when analysing differences between species. Significant differences between groups were tested with a Tukey Post Hoc test.

For the *Sphagnum* species we also had data on traits, and for these we analysed regressions with N_2_ fixation rates as the response variable, and decomposability in the lab, decomposition in the field, biomass growth, photosynthetic capacity and tissue element concentrations of biochemical compounds, C, N, P and K (as well as C/N and N/P ratios) as predictors. Residuals were checked for normality and N_2_ fixation rates were log-transformed (ln(y+1)). To analyse differences in nutrient concentrations, decomposition and growth parameters between samples of *S*. *magellanicum* from different habitats a nonparametric Kruskal Wallis test was used. Statistical tests were performed using SPSS Statistics 21.0 [44] and R version 3.4.0 [45].

## Results

### N_2_ fixation rates of *Sphagnum* and feather mosses from different habitats

**Fig 1:**
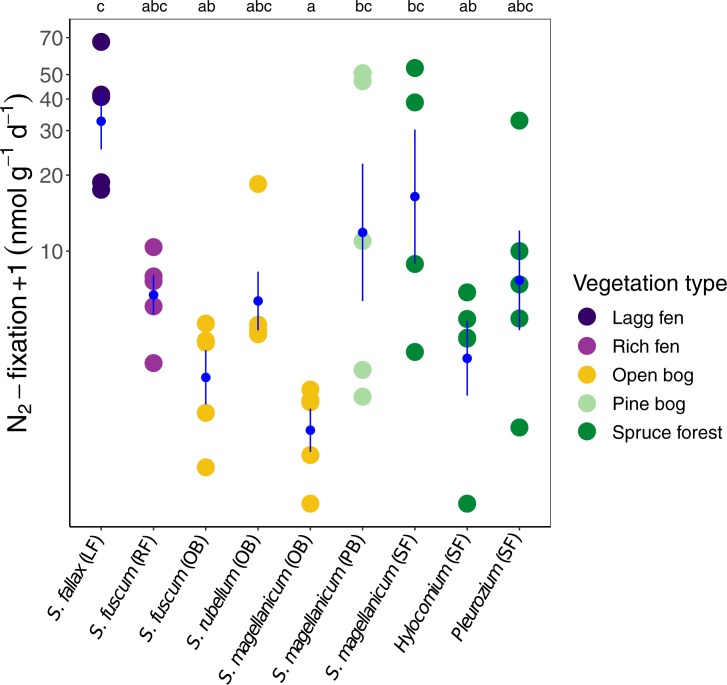
N_2_ fixation rates +1 on a logarithmic scale plotted for *Sphagnum* and feather moss species and habitats. Blue shows averages ± SEM (N = 5, except for *S*. *magellanicum* (SF) with N = 4). Different letters represent significant differences between species and habitats (based on Tukey tests).

N_2_ fixation rates varied between *Sphagnum* species (ANOVA: F_8,35_ = 5.35; P < 0.001, Fig 1). N_2_ fixation rates were the highest for *S*. *fallax* samples, but the rates of these samples could only be statistically distinguished from *S*. *fuscum* (OB), *S*. *magellanicum* (OB) and from the feather moss *Hylocomium splendens*. Compared with all *Sphagnum* samples, N_2_ fixation rates in the two feather mosses were at intermediate levels.

For *S*. *magellanicum*, we saw a strong effect of habitat (Fig 1) with N_2_ fixation averages being more than 20 times higher in the pine bog (PB) and spruce forest (SF), than in the open bog (OB). For this species, there were also significant differences in P concentration between the three habitats (Kruskal Wallis; P < 0.005): lowest P concentrations in OB, followed by PB and highest in SF.

N_2_ fixation could potentially be affected by microsite wetness (affecting redox potential), but we found no such relationship: the wettest carpet and lawn habitats had samples with both the highest (*S*. *fallax*) and lowest (*S*. *magellanicum*, open bog) rates (Fig 1), and the hummock samples were intermediate.

### Traits related to N_2_ fixation in *Sphagnum*

**Fig 2:**
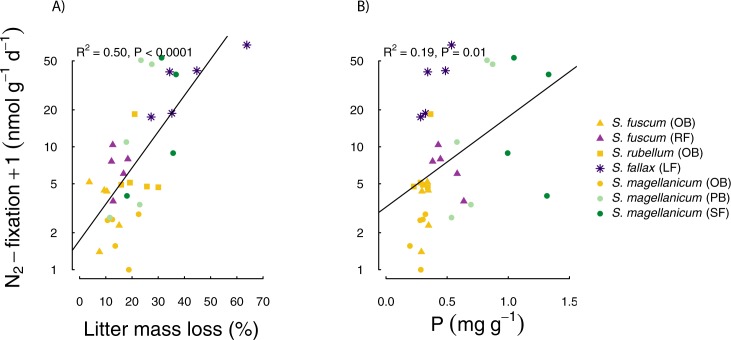
**N_2_ fixation rate +1 on a logarithmic scale, plotted against A) decomposability (litter mass loss (%) after 7 months incubation in the lab) and B) P concentration (mg g^-1^) of *Sphagnum* dry weight**.

N_2_ fixation rates for *Sphagnum* species throughout different habitats showed the strongest significant positive correlations with lab decomposition (r = 0.71; Fig 2A), and in order of weaker correlations with N/P ratio (r = -0.48), lignin-like phenolics (r = -0.46), P concentration (r = 0.43, Fig 2B), field decomposition (r = 0.40) and C concentration (r = -0.39) (Table 2). Photosynthetic capacity was most strongly correlated with P (r = 0.58), N (r = 0.51) and C/N (r = -0.43).

**Table 2:** Correlations within the *Sphagnum* data set.

	Loss lab	Loss field	Biomass growth	Photos. cap	C	N	P	K	N/P ratio	C/N ratio	Sphagnan	Soluble phenolics	Lignin-like phenolics
Nfix log	0.71**	0.4*	0.31	0.14	-0.39*	0.25	0.43*	0.30	-0.48**	-0.29	0.04	-0.17	-0.46**
Loss lab		0.45**	0.61**	0.22	-0.52**	0.31	0.31	0.02	-0.25	-0.33	-0.37*	-0.44*	-0.78**
Loss field			0.32	0.05	-0.24	0.47**	0.15	0.22	-0.02	-0.5**	-0.06	-0.08	-0.49**
Biomass growth				0.01	-0.39*	0.17	-0.14	-0.39*	0.29	-0.19	-0.50**	-0.60**	-0.43*
Photosynthetic capacity					-0.16	0.51**	0.58**	0.24	-0.33	-0.43*	0.04	-0.01	-0.09
C						-0.27	-0.19	0.02	-0.04	0.28	0.33	0.69**	0.59**
N							0.66**	0.43*	-0.14	-0.98	0.12	-0.10	0.01
P								0.60**	-0.77**	-0.63**	0.19	0.01	-0.06
K									-0.54**	-0.44**	0.26	0.39*	0.05
N/P ratio										0.14	-0.24	-0.23	0.10
C/N ratio											-0.13	0.10	0.01
Sphagnan												0.44*	0.53**
Soluble phenolics													0.53**

Variables are Nfix log = N_2_ fixation (log transformed (ln(y+1)) (nmol N_2_ g^-1^ h^-1^), Loss lab = decomposability, i.e. mass loss (%) from litter in lab conditions 7 months, Loss field = mass loss (%) from litter in field conditions 14 months, Biomass growth = biomass increase per area, averaged between the growth seasons of 2012 and 2013 (g cm^-2^), Photosynthetic capacity per unit dry weight (mg g^-1^ h^-1^), C, N, P, K = element concentrations (mg g^-1^) and N/P and C/N = nutrient ratios in *Sphagnum* tissue, and metabolites in *Sphagnum* tissue, all in mg g^-1^: sphagnan concentration, soluble phenolics concentration, and lignin-like phenolics concentration. *n = 31–34*. *, *P < 0*.05; **, *P < 0*.*01*.

In addition to the simple correlations (Table 2) we tested whether regression models with two predictors could predict N_2_ fixation better. The best combination (highest R^2^_adj_) was with decomposability and N/P ratio as predictors (ln(N_2_fixation rate +1) = 1.715 + 0.06 decomposability—0.055 N/P; P < 0.0001; R^2^_adj_ = 0.57; compared to R^2^_adj_ = 0.52 for the model with decomposability and P, and R^2^_adj_ = 0.49 for the simple model with decomposability as predictor).

N_2_ fixation was correlated with the lignin-like phenolics (Table 2), but adding the lignin-like phenolics to the regression model with decomposability did not increase the variation explained (R^2^_adj_ = 0.47). Testing models with three predictors did not improve on R^2^_adj_.

## Discussion

### Variation in N_2_ fixation rates

The potential N_2_ fixation rates we measured in feather mosses were on average 3.5 nmol N_2_ g^-1^ d^-1^ for *Hylocomium splendens* and 10.5 nmol g^-1^ d^-1^ for *Pleurozium schreberi*. This is within the range measured in boreal forests in Finland by Leppänen et al. [29]. They compared N_2_ fixation for the same two feather mosses in northern and southern (comparable to the latitude for our sites) habitats and found a northern average of around 27.9 nmol g^-1^ d^-1^ for both species, and a southern average of 1.8 nmol g^-1^ d^-1^ for both species. Lower rates in the southern sites were suggested to be linked to higher N deposition loads at lower latitudes in Scandinavia (> 0.3 g N m^-2^ y^-1^ compared to < 0.2 g N m^-2^ y^-1^) [29].

If we compare N_2_ fixation rates in feather mosses to rates in *Sphagnum* they are intermediate; the open bog sphagna tend to have lower rates, while mire margin sphagna tend to have higher rates. Higher rates in *Sphagnum* may be expected because of the more consistently higher water content of *Sphagnum*, leading to more anoxic and thus more favourable conditions for diazotrophs residing in the hyaline cells of *Sphagnum* (even though we did not find any relationship between habitat wetness and N_2_ fixation in the *Sphagnum* samples). The diazotrophic communities of *Sphagnum* species, mainly comprising Proteobacteria [25, 27], may be more dependent on wetter conditions to prevent oxygen from down regulating hydrogenase, than cyanobacteria, the main diazotrophs in the studied feather mosses [46]. This may lead to some *Sphagnum* species with higher and some with lower N_2_ fixation rates than in the feather mosses.

Studies about N_2_ fixation often include an up-scaling to potential annual fixation per unit area. Even though this is questionable in our case with measurements at only one occasion, we did some rough comparisons based on literature data. Annual N_2_ fixation rates are known to vary as a result of temperature and precipitation [46, 47], and are appreciable from June to September in Scandinavia [31, 48]. Assuming a growth season of 200 days [48], rates of annual N_2_ fixation in *Sphagnum* species in our study range on average from 6 to 120 mg N m^-2^ y^-1^. These rates are lower than results from other European peatlands of 140–280 mg N m^-2^ y^-1^ [11, 37], and much lower than 480–6230 mg N m^-2^ y^-1^ in boreal bogs in Canada [10]. However, at the moderate atmospheric N deposition rates in the research area (600 mg m^-2^ y^-1^; [49], inputs of on average 60 mg m^-2^ y^-1^ for *Sphagnum* species still represent an appreciable contribution.

### Drivers of *Sphagnum*-associated N_2_ fixation

Our most important finding is that N_2_ fixation rates of *Sphagnum* species were best explained by decomposability with an additional effect of phosphorus concentration (as P or N/P-ratio). Sphagna that produce higher amounts of compounds such as sphagnan and phenolics have higher intrinsic decay resistance (lower mass loss in the lab) [19], which was supported by the correlations found here. This may suggest that such compounds directly hamper diazotrophic activity. However, there could also be an indirect effect through decomposition rates and the resulting availability of P.

N_2_ fixation rates in mires have been found to be higher in wetter habitats [11, 37]. This was reflected by our finding that *S*. *fallax* consistently showed the highest rates of N_2_ fixation. However, *S*. *magellanicum* from the open bog, which grows equally wet, had relatively low N_2_ fixation rates. The large differences within *S*. *magellanicum* with lower rates on the bog than in the margin habitats, and overall low rates on the bog, suggest that N_2_ fixation rates are, at least partly, driven by differences between habitats (open bog versus mire margin). This also supports the old results (based on acetylene reduction assays) that open bogs have low rates of N_2_ fixation (e.g. [50]).

For *S*. *magellanicum*, different habitats were sampled, and we found the highest N_2_ fixation rates in the spruce forest. Since N_2_ fixation in *Sphagnum* is known to be much lower in darker conditions [36, 42], as would be the case under a canopy, light availability cannot explain the differences between open and treed habitats. Trees, assimilating nutrients from deeper soil layers and filtering dry deposition from the atmosphere provide input of nutrients as throughfall or litter [51]. Especially P has been shown to limit N_2_ fixation [3] and therefore a tree-covered habitat with higher P availability may stimulate N_2_ fixation rates as indicated for *S*. *magellanicum* in treed habitats (Fig 2B). The diazotrophic community of *S*. *magellanicum* from different habitats may not only differ in activity, but also in their composition [25]. It would therefore be interesting to know whether there is a direct microbiome link to different habitats [52].

Several variables were correlated with N_2_ fixation rates, and in the absence of controlled experiments, the mechanisms and directions of cause and effects cannot be fully clarified. Some predictors were also correlated with each other. For example, N_2_ fixation was negatively correlated with C, but C was also correlated with decomposability (Table 2), and did not improve on a model with decomposability as predictor for N_2_ fixation.

The positive correlation between N_2_ fixation and P (and negative with N/P ratio) may indicate that P increases the diazotrophs’ need for N to build up cell structures, instigating them to invest in N_2_ fixation as a source of additional N. This role of P in N_2_ fixation has been suggested before [3, 36, 39]. N_2_ fixation could also lead to more N in the *Sphagnum* tissue, which may in turn promote decomposition. *Sphagnum magellanicum* samples from the spruce forest have much higher average P concentrations than other species in our study, and the *S*. *magellanicum* samples from the pine bog lie in between (Fig 2B). Thereby, P concentrations in our data reflect different levels of P availability in the different habitats. The higher decomposition rates in more nutrient rich habitats may, in addition, provide a positive feedback on nutrient availability, as they increase nutrient turnover rates.

### N_2_ fixation and *Sphagnum* growth

We did not find any direct correlations between N_2_ fixation, photosynthetic capacity and growth (Table 1). Instead, higher *Sphagnum* growth is related to lower amounts of decay-resisting compounds, in accordance with the trade-off between growth and decomposition we found in a larger data-set [19, 32; of which the current data are a subset]. These previous studies also showed that growth is strongly modulated by, for example, water availability, and this may be more important than any direct effects of photosynthetic capacity or N_2_ fixation.

The *Sphagnum* species in our study showed N/P ratios indicating strong to moderate N limitation [53]. *Sphagnum magellanicum* from the spruce forest had the lowest value (9; indicating strong N limitation) and the same species had the highest value in the open bog (31; P limitation) (S1 Table and S2 Table). Toberman et al. [54] recognised that a key role for P was to drive productivity in peatlands, and these systems have been suggested to be co-limited by N and P, and possibly also by K [55–57]. Fritz et al. [55] showed that addition of P to a pristine bog lead to increased *Sphagnum* growth without a decrease in tissue N concentration. This may be explained by increased N_2_ fixation as a result of higher P availability. The importance of P as a regulator of microbial activity is also indicated by the high abundance of microbial genes for P uptake and transport found in *Sphagnum* and peat [58]. This shows that P, in addition to having a direct stimulating effect on *Sphagnum* as a nutrient co-limiting growth, can also have an indirect effect on *Sphagnum* growth through higher fixation of N.

There was a positive correlation between photosynthetic rate and tissue N concentration, which can be a result of higher nutrient input, faster mineralisation rates, higher N_2_ fixation rates, or a combination of these. The tissue N concentration included the N in the microbiome, so we cannot distinguish between the different sources of N. However, since N_2_ fixation is an energetically costly process, it is likely that the N being fixed is immediately used for the production of cell structures of diazotrophs (and other microorganisms), before becoming available for *Sphagnum* [36]. Consequently, in the longer term higher P availability also results in higher N availability, which would stimulate growth and influence the C sequestration function of peatlands. Whether changes in these microbial processes, as affected by P, result in a change in the activity or in the composition of these communities warrants further investigation.

### Synthesis: Towards a conceptual model

**Fig 3:**
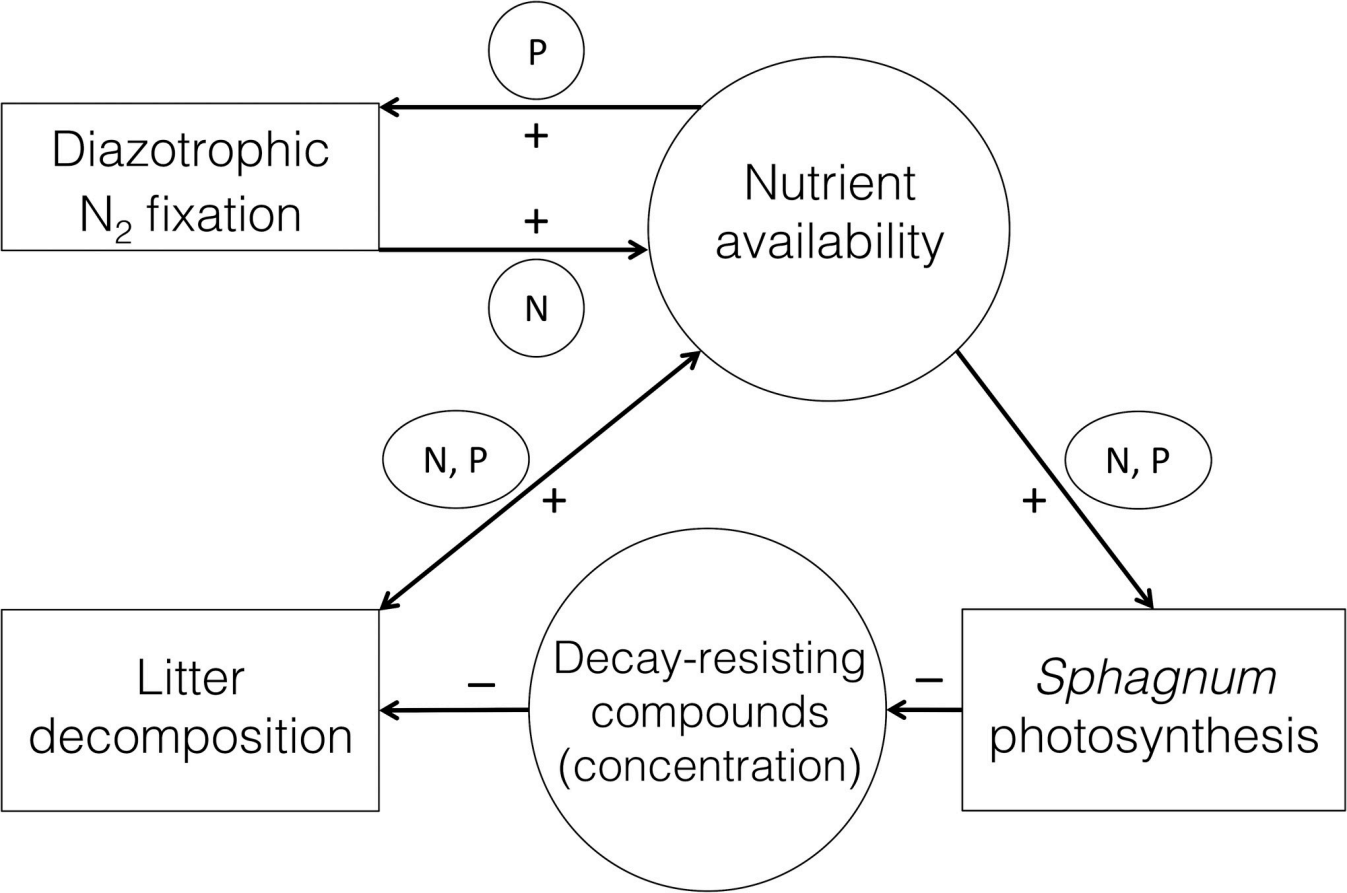
Conceptual graph showing the potential drivers of N_2_ fixation, *Sphagnum* photosynthesis and decomposition. Arrows indicate an effect (+ positive or–negative) of one process (squares) or reservoir (ellipses) on another, through the availability of nutrients or metabolites.

Based on literature and our findings, a conceptual model was generated that summarises the most probable drivers of N_2_ fixation, photosynthesis and decomposition in *Sphagnum*, the three processes that we measured in standardised laboratory conditions (Fig 3). This conceptual model may serve to generate testable hypotheses for experimental work in the future.

*Sphagnum* growth is generally limited by N or P, and in our results photosynthetic capacity was correlated with the concentration of both N and P. In contrast, N_2_ fixation was correlated with P. *Sphagnum* species-specific traits are determined by the trade-off between growth and decomposition. Decay rates are related to the concentration of metabolites inhibiting decomposition and to environmental factors such as pH, water table and nutrient availability. We here suggest that faster decomposition can lead to higher P availability (reaching the symbionts by the upward capillary water movement, or by active internal transport; cf. Rydin and Clymo [59]), while higher concentrations of decomposition-inhibiting metabolites may directly or indirectly impede N_2_ fixation.

Nutrient availability is governed by many processes not depicted in Fig 3 (such as other biological processes, nitrogen deposition, or nutrient inflow into fens and forested ecosystems). We speculate that if a higher input of P to the system (or habitat) stimulates N_2_ fixation rates, long term *Sphagnum* growth increases through increased N availability. In conclusion, higher availability of both N and P can be expected to result in increased turnover rates, resulting in a positive feedback loop.

## Supporting information

S1 Table Data used in the study. (CSV)

S2 Table Metadata for S1 Table. (CSV)
